# Prevention in practice – a summary.

**DOI:** 10.1186/1472-6831-15-S1-S12

**Published:** 2015-09-15

**Authors:** Stephen Birch, Colette Bridgman, Paul Brocklehurst, Roger Ellwood, Juliana Gomez, Michael Helgeson, Amid Ismail, Richard Macey, Angelo Mariotti, Svante Twetman, Philip M Preshaw, Iain A Pretty, Helen Whelton

**Affiliations:** 1 Centre for Health Economics and Policy Analysis, McMaster University, Canada; 2 Manchester Centre for Health Economics, University of Manchester, UK; 3 Public Health England, 4th Floor, 3 Piccadilly Place, London Road Manchester, M1 3BN, England, UK; 4 NWORTH, Y Wern, The Normal Site, Bangor University, Holyhead Road, Gwynedd, UK; 5 The Colgate Palmolive Company, Piscataway, NJ, United States; 6 Dental Health Unit, School of Dentistry, University of Manchester, Manchester, England, UK; 7 Dental Health Unit, School of Dentistry, University of Manchester, Manchester, England, UK; 8 Apple Tree Dental, 8960 Springbrook Drive, Suite 150 Minneapolis, MN 55433, USA; 9 Maurice H. Kornberg School of Dentistry, Temple University, Philadelphia, USA; 10 School of Dentistry, University of Manchester, Manchester, England, UK; 11 Division of Periodontology, College of Dentistry, The Ohio State University, Columbus, Ohio, 43210, USA; 12 Department of Odontology, Faculty of Health and Medical Sciences, University of Copenhagen, Copenhagen, Denmark; 13 School of Dental Sciences and Institute of Cellular Medicine, Newcastle University, Newcastle upon Tyne, NE2, 4BW, UK; 14 Dental Health Unit, School of Dentistry, University of Manchester, Manchester, England, UK; 15 School of Dentistry, University of Leeds, Leeds, LS2 9LU, UK

## Abstract

**Background:**

This paper is a summary document of the Prevention in Practice Conference and Special Supplement of BMC Oral Health. It represents the consensus view of the presenters and captures the questions, comments and suggestions of the assembled audience.

**Methods:**

Using the prepared manuscripts for the conference, collected materials from scribes during the conference and additional resources collated in advance of the meeting, authors agreed on the summary document.

**Results:**

The Prevention in Practice conference aimed to collate information about which diseases could be prevented in practice, how diseases could be identified early enough to facilitate prevention, what evidence based therapies and treatments were available and how, given the collective evidence, could these be introduced in general dental practice within different reimbursement models.

**Conclusions:**

While examples of best practice were provided from both social care and insurance models it was clear that further work was required on both provider and payer side to ensure that evidence based prevention was both implemented properly but also reimbursed sufficiently. It is clear that savings can be made but these must not be overstated and that the use of effective skill mix would be key to realizing efficiencies. The evidence base for prevention of caries and periodontal disease has been available for many years, as have the tools and techniques to detect, diagnose and stage the diseases appropriately. Dentistry finds itself in a enviable position with respect to its ability to prevent, arrest and reverse much of the burden of disease, however, it is clear that the infrastructure within primary care must be changed, and practitioners and their teams appropriately supported to deliver this paradigm shift from a surgical to a medical model.

## 

### Introduction

The proceeding papers in this special edition have described the individual elements required to place prevention at the heart of practice. From the definition and detection of common oral disease, the historical development of prevention, through to the economics and implementation of services, models and systems to promote prevention the proceeding papers provide a wealth of evidence.

Following the conference the authors and presenters wished to develop a document that distilled the information into a single collective summary. This work therefore broadly follows the outline of the conference, covering the following three main areas:

1. What is oral health and how can it be maintained?

2. How can we detect disease processes in a timely fashion so that prevention can work?

3. How can we organise dental services to support a transition from a surgical to a medical model, from care to cure.

When considering each of these three broad themes, the workshop groups were tasked with ensuring that the focus remained on what could be achieved within a community based general dental practice.

It is important to note that this summary represents a distillation of the articles provided in the supplement and also reflects on comments provided by attendees to the conference as well as discussion between the authors. References to main points are included, but the original papers contain the necessary citations to support the approach.

## 1) What is oral health and how can it be maintained?

### 

#### Definition of oral health

As a discipline, dentistry has focused on disease progression and the symptoms or disease history of a patient, rather than the maintenance of their oral health. The definition of oral health is difficult but will be defined as being the “absence of oral disease activity or progression with perceived well-being and without functional impairment". Such a definition is consistent with earlier categorizations such as that of the World Health Organisation [1]. As dentistry develops new and more powerful surrogate tests to enhance our understanding of oral health and disease, this definition is likely to evolve further.

#### How do we maintain oral health? The importance of self-care

The primary responsibility of maintaining oral health lies with the individual, or their custodians [2][3]. This should be achieved through effective; evidence based self-care but also needs to be supported by public health policy, education, the provision of professional monitoring and therapeutic interventions when necessary. Self-care is most effective when individuals are oral health literate and demand a dentition that is functional and aesthetically appealing. The dental care industry has an important role to play in the provision of effective, economic and accessible products to enable self-care. Maintenance of oral health is largely achieved through tooth brushing to provide plaque control (for gingival and periodontal health) combined with fluoride toothpaste (for caries prevention and treatment) [4]. Self-care should also be supported by a healthy diet, refraining from excessive alcohol intake or use of tobacco and regular visits to a dental professional to assess any disease activity or increased risk that may indicate change is required.

#### Public health policy

Public Health policy supports healthy environments and access to care to provide both prevention and reparative interventions that are effectively remunerated, encourage oral health literacy and individual empowerment. The numerous models of provision of oral care available whether they are free at point of delivery, insurance based or government supported may all be adapted to provide these basic prerequisites. In defining public health policy we must ensure that the benefits of oral health are understood and promoted by all, and that messages are clear, simple and above all consistent. Recent reports suggest that the profession is still unclear about simple oral hygiene measures and such variability in advice leaves patients confused about their role in securing and maintaining their oral health [5]. There may be a good case for dental organizations, such as IADR or FDI to develop and agree simple, consistent, evidence based guidelines for oral hygiene.

#### Professional care - a model of medical dentistry

The role of the dental care professional is to monitor oral health and assess risk of disease progression in addition to the more widely perceived role of provision of reparative surgical interventions. The monitoring of oral health and the provision of appropriate interventions when evidence of risk of disease progression is identified primarily achieves this. Once disease is identified this must be monitored to assess the success of any intervention through appropriate recall strategies. It is recognized that this medical model of dentistry, while promoted for many years and supported by strong research evidence, is not always at the center of dental training programmes and many dental care professionals still graduate as proficient surgeons, but lack the skills and knowledge to implement effective prevention [6].

#### Dominant Strategies

Given the definition of oral health provided above, and the roles and responsibilities of key stakeholders in securing and maintaining oral health what are the main evidence based strategies to prevent and treat disease when self-care fails? The main strategies are summarized in Table 1 together with the quality of evidence to support them. It is emphasized that the quality of evidence needs to be distinguished from the level of anticipated benefit and the strategies represent the current state of the art on disease control and prevention. The appropriate strategies for the individual are dependent on the level of risk and disease activity.

**Table 1:** Preventive Strategies to restore health

Category	Strategy	Mode	Caries	Erosion	Dentine Hypersensitivity	Periodontal & Gingival
**Fluoride**	Fluoride Toothpaste	SC	++++	+		

	High Fluoride Toothpaste and gels	SC/PC	++			

	Fluoride Varnish	PC	+++	+		

**Oral Hygiene**	Tooth brushing	SC			+	

	Flossing	SC				+

	Professional Tooth Cleaning	PC				++++

**Sensitivity reduction**	Toothpaste	SC			+	

**Diet**	Sugar Control	SC	+			

	Acid Control			+		

**Anti Bacterial**	CHX	SC/PC				

	Triclosan	SC				++++

	SnF	SC				++

Quality of evidence

++++ strong

+++ moderate

++ limited

+ insufficient

SC = Self care

PC = Professional care

#### Pre-requisites

The strategies defined in Table 1 are based on the detection of disease at a sufficiently *early stage* to enable maximum benefit. If the disease process is too advanced, for example gross caries into pulp with periapical infection, or advanced bone loss with highly mobile teeth - preventive therapies have little role in management. Community dental practitioners have generally been focused on the detection of disease at a stage where surgical intervention is required. If prevention in practice is to show real health benefits there needs to be a paradigm shift in diagnostic skills and approach to ensure that the disease processes are detected at a stage where medical interventions can be effectively implemented. Detecting disease at this level can be complex - and often times it may be difficult to differentiate from health.

## 2) How can we detect disease processes in a timely fashion so that prevention can work?

The previous section considered the definition of disease, the nature of the role of self and professional care and the dominant strategies available to prevent and arrest early stage oral diseases. However, in order to be able to enact these therapies effectively - diseases need to be detected, diagnosed, staged and the risk of the patient assessed. The Cape Town conference concentrated on the major oral diseases of caries and periodontal diseases, however, for completeness we have also covered, briefly, tooth surface loss and oral cancer.

### Dental Caries

#### Dental caries is a major cause of functional impairment in humans

Dental caries is the most prevalent condition afflicting humans [7] and has significant impact on quality of life, pain, and may even lead to life-threatening infections [8]. Dental caries is a complex multifactorial disease caused by an ecological shift in the microbiome following long-term frequent exposure to sugary or carbohydrate-containing drinks, foods, or snacks, poor oral hygiene practices and preventive care. Hence, to prevent the development of new caries lesions on sound tooth surfaces (primary prevention) and to stop the progression of early or initial caries lesions (secondary prevention), dental practitioners must first stage the caries process and assess its risk factors [9].

#### Staging of the caries progress is a pre-requisite to prevention

**Figure 1:**
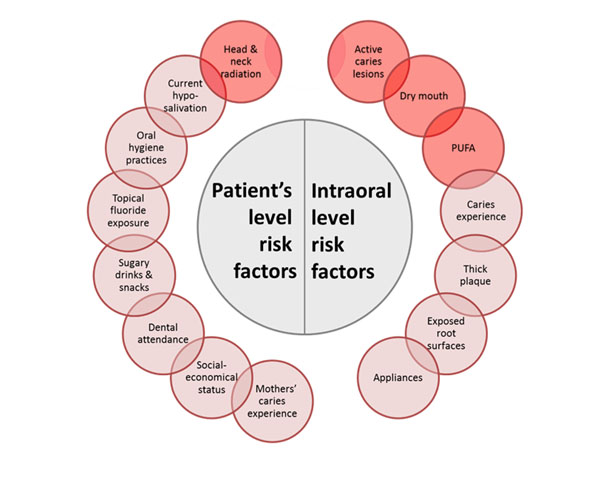
Risk factors associated with development of dental caries.

Appropriate management of dental caries requires diagnosing non-cavitated lesions in enamel and the outer one third of dentine [10]. These lesions can be prevented from progressing through risk management and targeted secondary preventive strategies [11]. Staging of caries may require both clinical and radiographic examinations. Clinical detection will require classification of teeth with active initial, moderate and extensive caries. Once lesions are classified an appropriate intervention can be developed. Table 2 presents this is in a simplified way - demonstrating the linkage of caries stage to treatment [12][13][4][14][15]. However the detection of lesions alone is insufficient to develop an effective preventive plan - lesions occur within patients as individuals and hence the assessment of risk factors and holistic treatment planning is essential. While a consensus on the most valid and easy to use risk assessment tool does not exist (and many are available), it is recommended, based on current evidence, that the risk factors shown in Figure 1 are considered [16][17][18][19][20] {Ritter, 2010 #10}.

**Table 2:** The combination of clinical and radiographic stages results in the following classification of the caries stages of teeth or tooth surfaces, as appropriate:

Sound tooth surfaces with no radiographic radiolucency.	Primary prevention
Initial or moderate clinical caries lesions and with initial radiographic stages of caries	**Control of progression**

Moderate or extensive caries lesions with moderate or extensive radiographic stages.	**Surgical treatment to promote function and aesthetics**

Red-circled factors are definitely associated with high caries risk. While other factors in combination or frequency, can collectively increase the risk of progression. For caries management decisions on behavioral and therapeutic interventions, patients’ risk status should be classified into low, medium or high. For each of these classifications, patients will receive different intensity and combination of clinical and personal self-care recommendations. As such patient centered care is developed further enhancing the integrated care model and moving away from the “one size fits all” surgical approach. It should be recognized that the use of risk based treatment planning is already advocated in many dental health systems - often associated with RAG ratings (Red, Amber Green). The International Caries Classification and Management System (ICCMS) is an excellent example of how this approach can operationalized and will provide a wealth of support material including caries management application and patient educational material [21]. It is also important to note that patients will, at different times, move between risk ratings - this might be due to short term changes in risk (for example pregnancy or an acute medical condition) or to longer term impacts such as the loss of manual dexterity or cognitive impairment.

### Periodontal Disease

Periodontal diseases comprise a group of highly prevalent chronic inflammatory conditions that affect the supporting structures of the teeth. The bacterial biofilm plays a fundamental role in initiating and perpetuating an inflammatory response in the periodontal tissues. Susceptibility to disease varies widely between individuals, dependent on environmental factors such as smoking, and genetic aspects of the immune and inflammatory functioning [22] Detection and diagnosis of periodontal conditions should be a routine component of oral and dental assessment.

#### Definition of periodontal diseases amenable to prevention

On the basis of our definition of oral health, the diagnosis of periodontal diseases infers progressing disease with functional impairment or pain in the absence of psychological or social well-being. Functional impairment in its broadest sense can include impaired function (e.g. chewing, speaking, socializing), pain (e.g. hypersensitivity), and/or compromised aesthetics. The periodontal conditions that will be considered include chronic periodontitis and gingivitis (i.e. plaque-induced gingivitis).

Chronic periodontitis is a bacterially induced chronic inflammation of the periodontal tissues that results in loss of attachment and alveolar bone destruction [23]. Progressing disease should be identified as part of regular dental assessment. Certain patients may have evidence of periodontitis which is stable (i.e. no evidence of progression), and that is not causing functional impairments. Gingivitis is bacterially induced inflammation that is confined to the gingival tissues. Utilizing our definition of health above, it could debated whether chronic gingivitis ever presents as a functional impairment, and at a histological level, gingival inflammation is evident in almost every person. On the other hand, certain patients may have anxieties regarding gingivitis, such as gingival bleeding, or aesthetic concerns. Recognizing that gingivitis is a proxy measure of poor oral hygiene and frequently precedes destructive periodontal involvement its detection should, however, remain an important element of assessing risk.

#### Staging of periodontal conditions

Like dental caries, it is important to stage and categorize periodontal conditions. In simple terms, we distinguish between gingivitis and periodontitis. Gingivitis is generally regarded as being reversible once inflammation reduces, and this is achieved by improved self-care and enabling professional care (often professional plaque removal). The tissue damage associated with periodontitis is largely irreversible, and therefore management focuses on prevention of periodontitis progression. Primary prevention of gingivitis should be a key aim of dental professionals, to maintain non-inflamed, healthy tissues. If gingivitis is identified, treatment should be provided to resolve the gingivitis, as this is a preventive strategy for preventing progression to periodontitis. If periodontitis is identified, management strategies should be implemented that aim to prevent further destruction of the periodontium.

#### Detection of disease

Initial visual inspection should be undertaken to identify evidence of gingivitis and level of oral hygiene. Periodontal screening should be performed utilizing the CPI (Community Periodontal Index) screening system (also referred to as the Periodontal Screening Record, PSR, and the Basic Periodontal Examination, BPE). CPI should be performed at every visit, and at least annually [24,25]. See Table 3.

**Table 3:** CPI Codes in periodontal assessments

CPI code 0	No increased probing depths, no bleeding on probing. Indicates healthy periodontal tissues.
CPI code 1	Bleeding on probing, together with clinical evidence of gingival inflammation (erythema, oedema), indicates gingivitis.

CPI codes 3 and 4	Probing depths of 4mm or greater, indicative of periodontitis. In this case, detailed periodontal examination should be performed, together with radiographic assessment of alveolar bone status as indicated by the clinical situation.

Assessment of periodontal status at a single point in time *cannot* provide information about disease progression. Therefore, assessments should be repeated over time to determine if attachment loss is progressing. However, many patients present with disease without the clinician being able to readily identify that the condition is progressing. Such patients may have a functional limitation, indicating a requirement for intervention. However, even in the absence of functional limitations or of proof of disease progression, periodontal disease in newly presenting patients does require intervention to prevent further progression.

#### Risk assessment to promote periodontal health

Periodontal disease is a complex, inflammatory disease that has multiple aetiologies, but a common clinical end-point. There is wide variation in susceptibility between individuals. The bacterial challenge perpetuates the chronic inflammatory response, which itself is influenced by environmental risk factors, the most important being smoking [26] and diabetes [27]. Assessment of risk should form part of the assessment of all patients. Key environmental risk factors to assess that are amenable to modification include:

• smoking behaviours: years of smoking, number of cigarettes per day, type of smoking, awareness of the impact of smoking on periodontal status, and interest to talk about quitting

• diabetes: level of glycaemic control (e.g. subjective assessments “good” vs. “poor”, or objective measures such as HbA1c), presence of other diabetes complications, awareness of the impact of diabetes on periodontal status

### Tooth surface loss

Tooth surface loss (TSL) may be purely physiological and occurs as a natural consequence of ageing [28]. Causative factors including erosion, abrasion and attrition can render tooth surface loss pathological. As a result of this, symptoms may develop and treatment may be indicated. As such TSL may contribute to a decline in oral health as defined within this work.

#### Erosion

Dental erosion is defined as a chemical process that involves the dissolution of enamel and dentine by acids not derived from bacteria when the surrounding aqueous phase is under saturated with tooth mineral. In contrast to caries, which develops as subsurface lesion body, dental erosion has been described as a surface phenomenon only. Clinically, the loss of structural integrity and mechanical strength is characterized by smoothness of the surface [29]. This process is followed by continuous layer-by-layer dissolution of enamel crystals, leading to a permanent loss of tooth volume with a softened layer at the surface of the remaining tissue. Erosive factors may be either intrinsic or extrinsic. Extrinsic sources include drinks such as fresh fruit juices, carbonated drinks, alcoholic beverages; and some foods and industrial processes. Intrinsic sources include gastro-oesophageal reflux and eating disorders, amongst others [29].

Currently erosion detection is based on visual identification [30]. Different clinical tooth surface loss indices have been used for epidemiological and clinical purposes. Recently the results of a workshop resulted in a new scoring system, the Basic Erosive Wear Examination (BEWE). This system was designed to provide a simple tool for use in general practice and to allow comparison to other more discriminative indices. It is a partial scoring system recording the most severely affected surface in a sextant with a four level score (Table 4) [31].

**Table 4:** The Basic Erosive Wear Examination (BEWE) index. [31]

Score	Description
0	No erosive tooth wear

1	Initial loss of surface texture

2*	Distinct defect, hard tissue loss, <50%of the surface area

3*	Hard tissue loss >50% of the surface area

While in the initial and moderate stages of erosion (BEWE 1), preventive or non-invasive strategies are indicated, in advanced stages (BEWE 2 and 3) more complex and invasive therapeutic strategies may be needed [32]. Determining factors for initiating therapeutics intervention are sensitivity, aesthetics and functional impairment [29].

### Oral cancer and premalignant conditions

Although not a study topic in this Symposium and considering the examination of preventive strategies for oral neoplasms involves extensive review that is beyond the scope of this Summary, there are several general comments concerning oral cancers requiring comment. Opportunistic cancer screening should be performed in every dental examination. Assessing patients for mouth cancer[29], and potentially malignant disease requires a high level of suspicion, but many other conditions may present with similar changes. The level of suspicion should be raised if the patient is a smoker or heavy alcohol drinker, chews betel nut (areca nut) or tobacco, or is over 40 years. More recently, the human papilloma virus (HPV), which is sexually transmitted, has been associated with cancers of the oral and oropharyngeal region. The screening for oral cancer would include detecting the presence or absence of malignant and potentially malignant lesions in the oral mucosa and distinguishing these from benign conditions.

## 3. How can we organize practice to deliver effective prevention?

### 

#### The current position

Primary care provision in dentistry has predominantly focused on the treatment of presenting oral disease in attending patients. This has largely arisen historically given the high of levels of disease present in the population over the last two to three decades. It has also been influenced by the way service reimbursement is linked to treatment interventions to serve demand [33], the way dentists are trained and how dental practices are organized to deliver treatment services [34] .

The consequences of this disease/treatment/demand focus are that:

1. attention becomes limited to dental treatment care (i.e. services are provided to meet presenting need [or demand] on an episodic basis by the dental care provider) as opposed to emphasis on self-care to prevent future disease, (i.e. the continuous attention given to explaining disease processes to promote healthy mouths involving daily self-care (oral hygiene, healthy diets) supported by regular periodic professional care.

2. as oral health improves, services are not being delivered in accordance with the relative needs (or relative risk of needs) or the distribution of these needs across different patient groups. Instead, the systems and processes of dentistry lock professionals into maintaining the status quo of service provision and responding to and inducing patient demands and supplier-induced demand.

3. there is incomplete coverage of the population by dental care provision beyond a stable patient base of regular attenders that not only fails to address social inequalities in oral health, but contributes to these inequalities.

4. there is a need for policy leaders to shift the focus from a traditionally surgical model of dentistry to an approach that concentrates on prevention by integrating mouth health with general health and social policies. Such an alliance will shift the focus in oral health from technical interventions towards social determinants of health and to greater equity and social justice.

These issues remain in many countries despite the use of different approaches to governing, delivering and funding dental care provision. This is largely due to the upstream nature of the social determinants of oral health and the downstream approaches to delivering care [35]. If untreated, dental disease can have a substantial impact on individuals’ health, functioning and self-esteem whilst also giving rise to school/work absence, reductions in productivity when at school/work and adverse effects on nutrition and development, particularly in children and adolescents.

#### Enablers for change

So what changes must take place in order to support the development of an effective prevention strategy for oral health? In the rest of this section we briefly identify and discuss the possible elements of such a policy organizing these under three broad headings, financial incentives, human resources and governance.

Financial incentives: Although many dentists are keen to adopt a preventive model of care, the systems in which they work often lead to such models not being structurally or financially viable. Where dentists rewards are based on what is done (throughput based) ‘in the here and now’ as distinct from who it is done for (population-based), the quality of care provided is naturally hampered [36] and engaging in prevention may lead to lower practice income [37]. By adopting alternative funding models that reward providers for engaging with hard to reach populations and high needs groups, we can ensure that from a provider perspective broadening the reach of their service pays appropriately [38][39].

In addition, practicing prevention has the potential to reduce service needs at both the individual patient and the population level, through a halo effect and hence reduce workloads at the practice level. This then frees up capacity to serve larger populations and further increases coverage. This virtuous circle approach must be tempered with the fact that if hard to reach groups are accessed, they may present with surgical need. Delivering this prior to the benefits of prevention being seen may impact on the perceived effectiveness of the approach - not least within the relatively short political cycles as compared to the relatively long disease processes of caries and periodontal conditions.

Human resources: The dentist may not be the most appropriate person to be delivering preventive care. Dental care professionals (DCPs) can be an effective and cost effective part of the dental care team within a prevention model. The adoption of an efficient ‘building’ design and skill-mix within a dental team provides an opportunity to use the team in a more productive way. Examples include dental nurses with additional competence and skills placing fluoride varnish and using motivational interviewing techniques, hygienists and therapists working to their full scope of practice to complete routine treatments and interim care preventive interventions in addition to screening examinations with a prevention focus in out-reach settings; enabling dentist time to be released to focus on more complex service needs and high risk/need populations [40].

The use of a wider skill mix within dental teams is in various stages of implementation globally - with some health systems readily accepting it - especially in areas where recruiting a more traditional dental workforce is problematic, but in others there has been significant push back from organized dentistry to resist the perceived erosion of the dentist as the center piece of oral care delivery [41]. By demonstrating the value of skill mix as an enabler for preventive care the ability for dentists to devote more resource to complex patients may be realized.

Patient responsibilities: Understanding the importance of personal homecare and eliminating destructive behaviors (e.g., smoking) become important factors for oral health. It is well known that both short-term improvement as well as long-term improvement in oral health can be obtained in patients but this must be individualized to the patient, otherwise, relapse will occur to baseline levels. To ensure acceptance and compliance, a significant aspect of care that immediately affects oral health is health literacy of the patient. Oral health literacy allows the patient to obtain services that are necessary for management of their oral health.

Governance: The delivery of effective prevention is a matter of public interest given the broad social impact of oral diseases on the one hand and the potential risk of harm from care provided by untrained or inadequately trained providers on the other. Policies are therefore required to support prevention in practice that (a) provide the appropriate regulatory framework to protect the public from harm and promote continual quality improvement in terms of training and competence of providers, (b) remove any impediments or restrictions on providers performing their full scope of practice, including but not limited to funding models that make adopting appropriate skill-mix in the financial interests of practice owners, not at the expense of those interests such as facilitating larger dental practice units that include preventive/ interview rooms in addition to surgeries.

### Summary

Overall the adoption of prevention in practice represents a paradigm shift in the way we think about oral health care, with the focus switching from intervention to a model that promotes health according to individual needs and risk. At a micro level changes are needed in the way that dentists approach the routine examination. There should be an assessment of individual risk and an investigation of the patient's current self-care routine and circumstances to establish a complete understanding of the patient's oral health needs and environment. This can be achieved by completing a needs assessment and creating a personalized daily self care plan for every patient.

This requires attention to be paid to the collection of data that relates to an individual's risks (hygiene, diet, tooth brushing habits) and needs (absence of pain and discomfort, functioning, self-esteem) as a basis for tailoring preventive care based on individual need. Excellent communication of the daily care plan is also essential. Practitioners should be motivated by the potential for increasingly rewarding relationships with patients which encourage them to manage and take responsibility for their own oral health and prevent disease rather than being dependent on the dentist to cure problems once they occur. Again, such an approach would lead to the potential of increasing the capacity to care for the wider population who currently do not attend and where much of the burden of oral disease resides.

Preventive practice should be further encouraged through ‘federated’ local networks to encourage dentists to reach out into their communities and integrate with their peers. This will ensure that services are “clinically led & clinically owned”, with strong leadership forming the basis for supporting effective service planning, quality improvement and engagement. This is not without its challenges. It requires a considerable shift in the culture of dentists and their team to share good practice and become motivated by the need of the local population rather than simply by the demand of their stable patient base. It also requires that policy leaders facilitate supportive environments, so that patients can adopt self-care messages and behavior change advocated by dental teams; as oral health inequalities result from social inequalities in the conditions of daily life.

### Conclusions

This special edition of BMC Oral Health was produced in response to a perceived “blockage” in the transfer of research into clinical practice. The evidence base has, for many years, increasingly supported the use of products, therapies and interventions that have been shown to prevent, arrest and reverse the commonest dental diseases that remain an issue globally. The development of diagnostic and staging systems and devices, combined with a recognition of the impact of risk on dynamic disease process, means that the disease processes can now be identified at an appropriate stage where they are amenable to early interventions. Despite this prevention in practice does not appear to be widely implemented.

The purpose of the Cape Town Conference was to collate the evidence for all three stages of prevention - identification of disease, the evidence based therapies and the enabling factors to implementation - together in a single document. It is clear that there are examples of best practice, where prevention has been instilled within services (See Bridgman, Helgeson in this supplement) despite existing and persistent barriers, but much more work is required, at all levels within the health care system to anchor prevention at the heart of dentistry. Governments, policy makers, insurers, organized dentistry, industry, the profession and patients all have a role to play in pushing clinical practice from surgical delivery of restorative treatments into a care model of health promotion and prevention.

## Declarations

This article has been published as part of *BMC Health Services Research* Volume 15 Supplement 1, 2015: Proceedings of Prevention in practice - making it happen. The full contents of the supplement are available online at http://www.biomedcentral.com/bmchealthservres/supplements/15/S1. Publication charges for this supplement were funded by Colgate Palmolive.

## References

[B1] Yewe-Dyer M The definition of oral health Br Dent J 1993 174 7 224 5 8461192 10.1038/sj.bdj.4808131

[B2] Sandberg GE Sundberg HE Wikblad KF A controlled study of oral self-care and self-perceived oral health in type 2 diabetic patients Acta Odontol Scand 2001 59 1 28 33 10.1080/000163501300035742 11318042

[B3] Marino R Albala C Sanchez H Cea X Fuentes A Prevalence of diseases and conditions which impact on oral health and oral health self-care among older chilean J Aging Health 2015 27 1 3 16 10.1177/0898264314533723 24850366

[B4] Walsh T Worthington HV Glenny AM Appelbe P Marinho VC Shi X Fluoride toothpastes of different concentrations for preventing dental caries in children and adolescents Cochrane Database Syst Rev 2010 1 CD007868 20091655 10.1002/14651858.CD007868.pub2

[B5] Wainwright J Sheiham A An analysis of methods of toothbrushing recommended by dental associations, toothpaste and toothbrush companies and in dental texts Br Dent J 2014 217 3 E5 10.1038/sj.bdj.2014.651 25104719

[B6] Iacopino AM The influence of “new science” on dental education: current concepts, trends, and models for the future J Dent Educ 2007 71 4 450 62 17468305

[B7] Petersen PE Bourgeois D Ogawa H Estupinan-Day S Ndiaye C The global burden of oral diseases and risks to oral health Bull World Health Organ 2005 83 9 661 9 16211157 PMC2626328

[B8] Bagramian RA Garcia-Godoy F Volpe AR The global increase in dental caries. A pending public health crisis Am J Dent 2009 22 1 3 8 19281105

[B9] Frencken JE Peters MC Manton DJ Leal SC Gordan VV Eden E Minimal intervention dentistry for managing dental caries - a review: report of a FDI task group Int Dent J 2012 62 5 223 43 10.1111/idj.12007 23106836 PMC3490231

[B10] Pitts NB Stamm JW International Consensus Workshop on Caries Clinical Trials (ICW-CCT)--final consensus statements: agreeing where the evidence leads J Dent Res 2004 83 Spec No C C125 8 15286139 10.1177/154405910408301s27

[B11] Ismail AI Tellez M Pitts NB Ekstrand KR Ricketts D Longbottom C Caries management pathways preserve dental tissues and promote oral health Community Dent Oral Epidemiol 2013 41 1 e12 40 10.1111/cdoe.12024 24916676

[B12] Marinho VC Worthington HV Walsh T Clarkson JE Fluoride varnishes for preventing dental caries in children and adolescents Cochrane Database Syst Rev 2013 7 CD002279 23846772 10.1002/14651858.CD002279.pub2 PMC10758998

[B13] Tellez M Gomez J Kaur S Pretty IA Ellwood R Ismail AI Non-surgical management methods of noncavitated carious lesions Community Dent Oral Epidemiol 2013 41 1 79 96 10.1111/cdoe.12028 23253076

[B14] Slot DE Vaandrager NC Van Loveren C Van Palenstein Helderman WH Van der Weijden GA The effect of chlorhexidine varnish on root caries: a systematic review Caries Res 2011 45 2 162 73 10.1159/000327374 21525751

[B15] Anderson CA Curzon ME Van Loveren C Tatsi C Duggal MS Sucrose and dental caries: a review of the evidence Obes Rev 2009 10 Suppl 1 41 54 19207535 10.1111/j.1467-789X.2008.00564.x

[B16] Pitts NB How the detection, assessment, diagnosis and monitoring of caries integrate with personalized caries management Monogr Oral Sci 2009 21 1 14 19494672 10.1159/000224208

[B17] Tellez M Gomez J Pretty I Ellwood R Ismail AI Evidence on existing caries risk assessment systems: are they predictive of future caries? Community Dent Oral Epidemiol 2013 41 1 67 78 10.1111/cdoe.12003 22978796

[B18] Ekstrand KR Bruun G Bruun M Plaque and gingival status as indicators for caries progression on approximal surfaces Caries Res 1998 32 1 41 5 10.1159/000016428 9438570

[B19] Ismail AI Sohn W Lim S Willem JM Predictors of dental caries progression in primary teeth J Dent Res 2009 88 3 270 5 10.1177/0022034508331011 19329463 PMC3317940

[B20] Leong PM Gussy MG Barrow SY de Silva-Sanigorski A Waters E A systematic review of risk factors during first year of life for early childhood caries Int J Paediatr Dent 2013 23 4 235 50 10.1111/j.1365-263X.2012.01260.x 22925469

[B21] Pitts NB Ekstrand KR Foundation I International Caries Detection and Assessment System (ICDAS) and its International Caries Classification and Management System (ICCMS) - methods for staging of the caries process and enabling dentists to manage caries Community Dent Oral Epidemiol 2013 41 1 e41 52 10.1111/cdoe.12025 24916677

[B22] Preshaw PM Taylor JJ How has research into cytokine interactions and their role in driving immune responses impacted our understanding of periodontitis? J Clin Periodontol 2011 38 Suppl 11 60 84 21323705 10.1111/j.1600-051X.2010.01671.x

[B23] Pihlstrom BL Michalowicz BS Johnson NW Periodontal diseases Lancet 2005 366 9499 1809 20 10.1016/S0140-6736(05)67728-8 16298220

[B24] Periodontology BSo, Basic Periodontal Examination 2011

[B25] Puri K Puri N Dodwad V Masamatti SS Restorative aspects of periodontal disease: an update part 1 Dent Update 2014 41 6 545 8 551-2 25195488 10.12968/denu.2014.41.6.545

[B26] Warnakulasuriya S Dietrich T Bornstein MM Casals Peidro E Preshaw PM Walter C Oral health risks of tobacco use and effects of cessation Int Dent J 2010 60 1 7 30 20361572

[B27] Preshaw PM Alba AL Herrera D Jepsen S Konstantinidis A Makrilakis K Periodontitis and diabetes: a two-way relationship Diabetologia 2012 55 1 21 31 10.1007/s00125-011-2342-y 22057194 PMC3228943

[B28] Wood I Jawad Z Paisley C Brunton P Non-carious cervical tooth surface loss: a literature review J Dent 2008 36 10 759 66 10.1016/j.jdent.2008.06.004 18656296

[B29] Lussi A Carvalho TS Erosive tooth wear: a multifactorial condition of growing concern and increasing knowledge Monogr Oral Sci 2014 25 1 15 24993253 10.1159/000360380

[B30] Attin T Wegehaupt FJ Methods for assessment of dental erosion Monogr Oral Sci 2014 25 123 42 24993262 10.1159/000360355

[B31] Ganss C Lussi A Diagnosis of erosive tooth wear Monogr Oral Sci 2014 25 22 31 24993255 10.1159/000359935

[B32] Schlueter N Jaeggi T Lussi A Is dental erosion really a problem? Advances in Dental Research 2012 24 2 68 71 10.1177/0022034512449836 22899683

[B33] Birch S The identification of supplier-inducement in a fixed price system of health care provision. The case of dentistry in the United Kingdom J Health Econ 1988 7 2 129 50 10.1016/0167-6296(88)90012-4 10288955

[B34] Glick M Monteiro da Silva O Seeberger GK Xu T Pucca G Williams DM FDI Vision 2020: shaping the future of oral health Int Dent J 2012 62 6 278 91 10.1111/idj.12009 23252585 PMC9374976

[B35] Watt R Sheiham A Inequalities in oral health: a review of the evidence and recommendations for action Br Dent J 1999 187 1 6 12 10452185 10.1038/sj.bdj.4800191

[B36] Tickle M McDonald R Franklin J Aggarwal VR Milsom K Reeves D Paying for the wrong kind of performance? Financial incentives and behaviour changes in National Health Service dentistry 1992-2009 Community Dent Oral Epidemiol 2011 39 5 465 73 10.1111/j.1600-0528.2011.00622.x 21668463

[B37] Grytten J Models for financing dental services. A review Community Dent Health 2005 22 2 75 85 15984132

[B38] Birch S Kephart G Murphy GT O'Brien-Pallas L Alder R MacKenzie A Health human resources planning and the production of health: development of an extended analytical framework for needs-based health human resources planning J Public Health Manag Pract 2009 15 6 Suppl S56 61 19829233 10.1097/PHH.0b013e3181b1ec0e

[B39] Whittaker W Birch S Provider incentives and access to dental care: evaluating NHS reforms in England Soc Sci Med 2012 75 12 2515 21 10.1016/j.socscimed.2012.09.035 23103073

[B40] Brocklehurst PR Tickle M Is skill mix profitable in the current NHS dental contract in England? Br Dent J 2011 210 7 303 8 10.1038/sj.bdj.2011.238 21475274

[B41] Nash DA Friedman JW Kardos TB Kardos RL Schwarz E Satur J Dental therapists: a global perspective Int Dent J 2008 58 2 61 70 18478885 10.1111/j.1875-595x.2008.tb00177.x

